# Progress in Biosensors for the Point-of-Care Diagnosis of COVID-19

**DOI:** 10.3390/s22197423

**Published:** 2022-09-29

**Authors:** Miroslav Pohanka

**Affiliations:** Faculty of Military Health Sciences, University of Defense, Trebesska 1575, CZ-50001 Hradec Kralove, Czech Republic; miroslav.pohanka@unob.cz

**Keywords:** antibody, antigen, coronavirus disease, diagnosis, handheld assay, immunosensor, lateral flow tests, lateral flow immunoassay, point-of-care test, SARS-CoV-2

## Abstract

Coronavirus disease 2019 (COVID-19) is a highly virulent infection that has caused a pandemic since 2019. Early diagnosis of the disease has been recognized as one of the important approaches to minimize the pathological impact and spread of infection. Point-of-care tests proved to be substantial analytical tools, and especially lateral flow immunoassays (lateral flow tests) serve the purpose. In the last few years, biosensors have gained popularity. These are simple but highly sensitive and accurate analytical devices composed from a selective molecule such as an antibody or antigen and a sensor platform. Biosensors would be an advanced alternative to current point-of-care tests for COVID-19 diagnosis and standard laboratory methods as well. Recent discoveries related to point-of-care diagnostic tests for COVID-19, the development of biosensors for specific antibodies and specific virus parts or their genetic information are reviewed.

## 1. Introduction

Coronavirus disease 2019 (COVID-19), caused by severe acute respiratory syndrome coronavirus 2 (SARS-CoV-2), emerged as an abrupt epidemic with a global impact and has been declared a pandemic by the World Health Organization since 11 March 2020. Since the pandemic began, many strategies have been established to prevent the spread of the disease. The use of protective means, the quarantine strategy, travel restrictions, and vaccination can be mentioned [1,2,3,4,5,6]. The efficacy of these approaches in establishing national strategy countermeasures to COVID-19 has been limited, and the countermeasures caused side effects, including a social impact on the population and economy [7,8,9,10]. The increased focus on COVID-19 also suppresses healthcare capacities for other diseases that can be manifested in other areas, such as cancer therapy and prevention [11]. 

Explicit disclosure of disease onset is one of the basic approaches in countermeasures to COVID-19 and reduce unspecific precautions that cause collateral damage and expenses. Since the pandemic started, great progress has been made in instrumental methods for the diagnosis of COVID-19, and new analytical methods have been invented. Point-of-care tests, biosensors and portable bioassays were recognized as crucial tools as capacity was restricted and some regions were not even equipped for performing instrumental diagnoses. The great progress of biosensors and point-of-care tests on COVID-19 diagnosis followed early after the onset of the pandemic [12,13,14]. 

Biosensors are analytical devices in which a biomolecule, also called a biorecognition element, is combined with a sensor transduction system, also called a physicochemical transducer. The outgoing signal in the form of a physical property is measured by an analyzer. 

This review focuses on the development of new biosensors suitable for the diagnosis of COVID-19 by the detection of specific parts of the viruses (or markers in the infected people and are suitable for testing at the point-of-care testing or in handheld assay conditions. The actual trends and literature on this issue are surveyed here. The review contains a comparison with the currently available point-of-care tests and discussion about limitations of various diagnostic approaches when COVID-19 was revealed. 

## 2. Current Point-of-Care Tests for COVID-19

COVID-19 is a viral respiratory disease with increasing mortality with increasing age of patients and occurring comorbidities such as obesity, diabetes and immune deficiency [15,16,17]. The disease has typical indicators of shortness of breath (dyspnea), fever, fatigue and cough, but other manifestations can occur depending on the variant of COVID-19 and individual dispositions, and some post-COVID-19 syndromes can remain even after the infection resolves [18,19,20,21,22,23]. An increase in inflammatory cytokines and the appearance of symptoms after inflammation is also typical for COVID-19 [24,25,26,27,28]. The exact diagnosis can be based on various techniques and instruments, including the techniques locating damaged tissues such as tomography and sonography [29,30,31] and molecular biology methods such as polymerase chain reaction (PCR) and loop-mediated isothermal amplification (LAMP) in their reverse transcriptase [32,33,34,35,36,37,38]. Immunochemical methods such as enzyme-linked immunosorbent assay (ELISA) for the detection of specific antibodies or the presence of the COVID antigen can also be used [39,40,41,42]. The mentioned methods generally exert good accuracy and reliability. On the other hand, common laboratory assays require reliable and expensive equipment and infrastructure, systematic quality control, instrument services and experienced and educated personnel to be performed [43,44,45,46,47]. During the COVID-19 pandemic, simple means of diagnosing the disease or detecting the presence of covid antigens or other markers were highly desired and were helpful when available in sufficient numbers and at a low price at the same time.

Lateral flow tests have become the main analytical tool for the diagnosis of COVID-19 since the early phases of the pandemic [48,49,50]. The lateral flow tests are a simple analytical tool that has spread since the 1980s. The first applications of lateral flow tests were focused on revealing but further analytes followed, and the tests gained popularity as tools for the analysis of biochemical and immunochemical markers, toxins, microorganisms, pollutants, drugs, pharmaceutics and others [51,52,53,54,55,56,57]. These devices resemble biosensors and some scholars consider them a type of biosensor. Compared to standard types of biosensors, lateral flow tests do not contain a physical sensor, as coloration is read by the naked eyes. On the other hand, readers of coloration are provided by some companies, and therefore lateral flow tests combined with colorimetric readers can be considered biosensors.

The principle of lateral flow tests is based on an affinity interaction between a recognition molecule immobilized on a matrix and another recognition molecule that is labeled by a dye, fluorescent label, colored or fluorescent nanoparticles or other molecule allowing visualization. The antibody is a typical recognition molecule used in lateral flow tests, but aptamers have also gained a high popularity for the purpose, and various antigenic and receptor structures are also suitable for test manufacturing [58,59,60]. During the assay, the analyte interacts with the labeled recognition molecule and migrates by capillary flow on the matrix at the same moment. Migration is stopped by capturing the complex-analyte-labelled recognition molecule by another recognition molecule of the analyte that is chemically bound to the matrix and forms the lines that the user of a test. The second line is typically used for controls and it contains an attached antibody or another molecule specific to the labeled recognition molecule. This is the reason why the control line is formed even if no analyte is present in the tested sample. The colored lines can be easily read by the naked eye without any equipment, and also the assay does not need any specific tools or sample processing; therefore, the lateral flow tests are suitable for point-of-care conditions as a simple and inexpensive method. In the linear dynamic concentration range of the assay, the color intensity of the lines is proportional to the analyte concentration [61]. On the other hand, the common tests are not designed or suitable for the exact determination of the analyte concentration, and they provide only qualitative information on the presence or absence of analyte in a sample, and the intensity of the color is a crude estimation only. Although some devices can be used for the estimation of analyte concentration by measuring the optical density of colored lines, they can be taken as a semiquantitative assay with limited accuracy. Quite high limits of detection are other drawbacks that are typically significantly higher for lateral flow tests than for standard laboratory immunoassays such as ELISA. 

**Figure 1:**
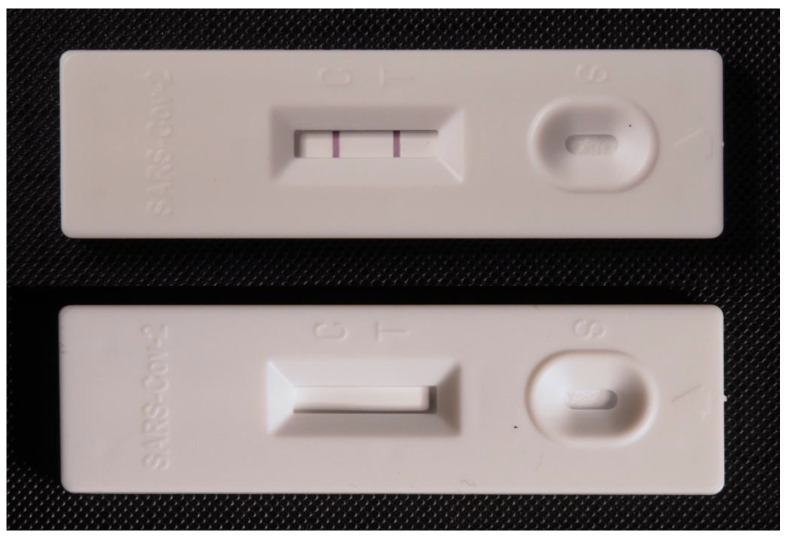
Example of a commercially available antigen lateral flow test for the diagnosis of COVID-19. A test with formed test and control lines is depicted in the upper photograph. An unused test is shown in the bottom figure.

Lateral flow tests play an important role in the early diagnosis of COVID-19 and both the determination of the respective antigens from the virion particles and antibodies of the particles can be measured by the commercially available tests. Common tests for COVID-19 diagnosis are based on the detection of specific structures from virion particles, and they are called antigen or antigenic tests; these tests are typically determined for analysis of nasopharyngeal swab samples. An example of a standard antigen lateral flow test for COVID-19 is depicted in Figure 1. They can be used in home care conditions or in field epidemiological screening, where they can confirm the manifested disease and reveal some phases of latent infection [62,63,64]. The sensitivity of the available test kits is generally significantly lower than the sensitivity of reverse transcriptase PCR [65,66] or ELISA [67], and false negativity can be easily concluded by lateral flow tests. The specificity of antigen lateral flow tests for COVID-19 is quite good [68]. Nevertheless, there can be issues with false positivity in some cases and some studies pointed to number of people falsely tested as positive [69]. 

## 3. Concept of Point-of-Care Biosensors for COVID-19 Diagnosis

**Figure 2:**
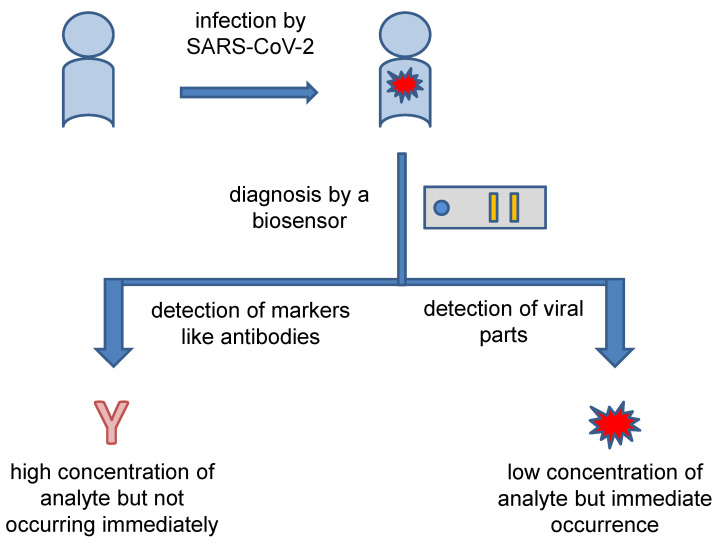
General concept of the diagnosis of COVID-19 by biosensors.

Taking into account the current methods for the diagnosis of COVID-19, the concept of a biosensor for diagnosis purpose can be based on either on direct detection of a determinant structure or indirect diagnosis based on the measurement of immunochemical markers created as a response to disease progression. The general concept is depicted in Figure 2. The indirect diagnosis based on measuring of immunochemical markers such as specific antibodies is easier from a technological point of view because specific antibodies (the most common marker for COVID-19) or certain cytokines (the less common marker for COVID-19) are released in a mass amount in the response to the disease, and they also exert chemical uniformity. The number of antibodies and cytokines is amplified considering the stimulus by the pathogen. In an instance, one B lymphocyte can produce a number of specific immunoglobulins (Ig) when stimulated by the pathogen. The response also remains for a certain period even after the erasing of SARS-CoV-2 from the blood. A lag between COVID-19 progression and the markers’ presence in the blood is a disadvantage of the indirect diagnosis. Specific antibodies against SARS-CoV-2 can be demonstrated approximately one week after the onset of the disease symptoms [70]. Specific IgG and IgM are probably the best markers for which new biosensors should be aimed when analyzing blood samples from suspected COVID-19. In a 2020 study, seroconversion times of 12 days for IgM and 4 days for IgG were reported [71]. However, the beginning of antibody production is somewhere between 1 and 2 days after infection starts though the amount of antibodies released is still low at this moment and the speed of antibody production is also individual depending on various factors [72]. A highly sensitive biosensor would prove the presence of Ig soon after infection, but a reliable diagnosis using antibodies is not expected in the early stages of COVID-19. In addition to the diagnosis of COVID-19 disease, the analysis of anti-SARS-CoV-2 antibodies serves to control vaccination efficacy or the evaluation of natural immunity against the disease, because vaccination has unequal efficacy in various groups, such as people with chronic diseases and dialyzed patients as compared to healthy people of working age [73,74,75,76,77]. Biosensors can be a functional tool for this supervision of vaccination efficacy. 

The direct SARS-CoV-2 assay from samples such as blood, nasopharyngeal swab samples and feces is unambiguous proof of the infection even before the onset of symptoms and confirmation of the COVID-19 disease when a symptomatic phase begins. Gargles and mouth wash are also suitable for sampling [78]. Membrane proteins (M), envelope proteins (E), spike proteins (S) and nucleocapsid proteins (N) and single-stranded positive-sense RNA with specific genes for the aforementioned proteins are the most important structures to analyze [79,80]. Biosensors and assays for direct detection of SARS-CoV-2 virions or its determinant structures should be highly sensitive because the concentration of typical markers is low. In addition, a large part of the viral particles is, moreover, closed within host cells [81]. In a study on the N protein of SARS-CoV-2, there was found a concentration of around 1 ng/mL in serum samples from patients with manifested disease, but infected patients with an asymptomatic phase can have N protein in the concentration of approximately 10 pg/mL [82]. A mean concentration of N protein in blood samples of 1.73 ng/mL was reported in another study [83].

When considering the principle of the biosensors for COVID-19 diagnosis, there is not a substantial difference in physical assay principle of the SARS-CoV-2 antigenic or other structure assays and assays for anti- SARS-CoV-2 antibodies. All basic types of biosensors, including electrochemical, optical and piezoelectric, can be adopted for the purpose of preparing a point-of-care diagnostic tests for COVID-19. 

Adaptation of the specific types of biosensors into praxis will depend not only on analytical specifications but also on expenses, which will play an important role in decisions for which point-of-care test will be preferred. Consumption of energy or raw material consumption can have a negative effect on the manufacturing costs. Prices of material and energy have become highly volatile, and some types of biosensors, such as electrochemical devices with electrodes manufactured from noble metals, could have problems being competitive. The production of metal nanoparticles used in both the electrochemical and optical biosensor can also be influenced by recent economic events and an increase in the price of the nanoparticles can be expected. The volatility of prices can also be related to the biorecognition element. Production of monoclonal antibodies is, for instance, a quite expensive process where energies are also necessary. However, the situation can change due to economic cycles and cheaper and more innovative mass production and the prices can drop again. 

## 4. Biosensors for Measuring Anti-COVID-19 Antibodies

Biosensors for diagnosis of COVID-19 by assay of anti-COVID-19 antigen-specific antibodies have a common sense for confirmation of specific antibody presence as a result of the disease or for measuring of seroconversion initiated by vaccination or the disease. The specific antibodies are not a tool for the diagnosis of an undergoing disease in an acute phase because specific antibodies are created after a delay of several days and highly specific antibodies such as IgG are presented in the blood even later. The first phases of an infection disease (incubation and prodromal) are typically not covered by antibody production, while the antibodies remain up to the convalescence phase of the disease and further while other biochemical and immunochemical markers gain their normal values. The practical relevance of biosensors as tools for point-of-care testing is lower compared to the COVID-19 antigens. However, such biosensors would improve the quality of care by faster and more accurate decisions for revaccination and identification of the most endangered people by the disease development. 

Cady and coworkers developed a multiplexed grating-coupled fluorescent plasmonic biosensor for the detection of antibodies specific to spike S1 protein, spike S1S2 protein, the and N protein as these proteins were immobilized on a biosensor chip [84]. This type of spectroscopic assay exerted 100% selectivity and sensitivity for an assay of specific IgG and selectivity of 100% and sensitivity of 87% for an assay of total anti-SARS-CoV-2 antibodies in blood samples from human volunteers. The other specific antibodies, including immunoglobulins IgM and IgA, can also be analyzed. The assay was also fully correlated with standard ELISA. In another application of a non-linear optical method, a surface plasmon resonance biosensor was developed for the assay of IgG, IgM and IgA specific to the receptor binding domain of SARS-CoV-2 [85]. The biosensor contained immobilized S protein and was verified on 102 human samples in which the presence of anti-S protein antibodies and their isotype were measured in a single step. The sensitivity of the test reached 90% for IgG, 95% for IgA and 98% for IgM, while the best specificity was achieved for the IgG assay at 98%, followed by IgA at 91% and IgM at 89%. Total sensitivity was equal to 93% and specificity to 100%. Though this biosensor exerted good properties, it is not a common example of a point-of-care test because of the price and size of the surface plasmon resonance principle. Maybe future development can reduce the price and make this platform more suitable for applications outside the laboratory environment. 

Nanoplasmonic biosensing platforms with immobilized S protein on gold triangular nanoprisms served as anti-SARS-CoV-2 antibodies in the work of Masterson et al. [86]. The device, working on the principle of non-linear optics, exerted a very low limit of detection equal to 30 amol/L for the specific IgG molecules, and 90% specificity and 100% sensitivity were proved when the device was tested on a group of 121 COVID-19-positive patients and 65 healthy individuals.

Bao and co-workers developed a simple colorimetric vertical flow immunoassay biosensor [87]. The colorimetric device was suitable for the recognition of IgG and IgM antibodies specific to S protein, and the visualization was done in presence of secondary antibodies labeled with gold nanoparticles on a nitrocellulose membrane that served as a matrix for the reaction. The whole assay was completed in 2 min. Although the principle of the test is quite common, the authors made a promising innovation based on the manner in which samples are taken. The bottom part of the biosensor was fabricated from biodegradable porous microneedles from polylactic acid that can safely penetrate the skin and collect interstitial fluid through the capillary effect. A point-of-care device for the detection of specific anti-SARS-CoV-2 antibodies was also developed by Mattioli et al. [88]. They immobilized a SARS-CoV-2 antigen (receptor binding domain bioconjugate) on a graphene-based electrode and verified the prepared biosensor on real human serum samples by voltammetry. The electrochemical device detected IgG specific to the receptor binding domain of SARS-CoV-2 with a limit of detection of 1.0 pg/mL for a sample sized 40 µL. An assay was finished within 15 min. All the specifications in combination with the fact that the biosensor was a miniaturized device makes it suitable for testing in point-of-care conditions. A miniaturized device with the potential to be used in point-of-care conditions was also constructed on a fluorometric principle [89]. The fluorometric assay was conceived as an optofluidic fluorescence biosensor where the S protein was the recognition part interacting with IgG in the tested sample. In the second step, an Alexa Fluor 680-labeled goat anti-human IgG secondary antibody was applied, and fluorescence was detected by fiber optics. Because the secondary antibody was specific to the IgG class of immunoglobulins, other immunoglobulins including IgM, were not detected during the analysis. It makes the assay more specific; on the other hand, it may cause lower sensitivity for the diagnosis of COVID-19 in the early phases. The specific IgG was proved with a detection limit of 12.5 ng/mL for a sample sized 100 µL and an assay lasting 25 min. Xu et al. constructed another fiber optic device for the detection of IgG and IgM against S protein [90]. The researchers opted for a Fresnel reflection microfluidic biosensor as a device allowing quite fast and label-free detection of the antibodies. The microfluidic chip in the device contained fiber with attached goat anti-SARS-CoV-2 IgG and IgM antibodies responsible for the interaction with the specific IgG and IgM from tested samples. The limit of detection for IgM antibodies against SARS-CoV-2 was equal to 0.82 ng/mL, while the assay of IgG specific to SARS-CoV-2 exerted a limit of detection of 0.45 ng/mL.

A field effect transistor voltammetric biosensor was developed for the detection of IgG anti-SARS-CoV-2 antibodies, but it was also suitable for antigen detection [91]. The electrochemical biosensor contained titanium-gold working electrodes improved by In_2_O_3_ nanoribbons, and the Ag/AgCl electrode served as a reference electrode. The electrochemical assay was based on the formation of an immunocomplex containing streptavidin and alkaline phosphatase. Alkaline phosphatase catalyzed the conversion of p-nitrophenyl phosphate to p-nitrophenol and the drop in pH causes protonation of In_2_O_3_ nanoribbon surface and increases material conduction. The limit of detection for IgG specific against S protein was equal to 1 pg/mL. 

An avidity testing-on-a-probe biosensor assay measuring platform was developed for COVID-19 diagnosis by Yang and coworkers [92]. The flurimetric assay was based on a quartz with S protein probe interacting with specific antibodies, another S protein with Cy5-Streptavidin-polysacchcaride conjugate was added after sample application and the fluorescence signal was recorded when the total neutralizing antibodies were analyzed. Taking into account the figures presented, approximately 2.5 µg/mL of IgG and IgM were recorded by the method. The assay was further adapted for an anti-COVID-19 antibodies assay [93].

**Figure 3:**
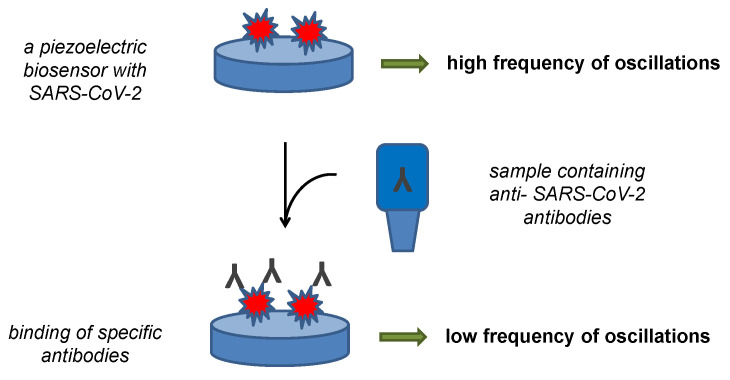
Principle of a piezoelectric biosensor for the detection of anti-SARS-CoV-2 antibodies.

Antibodies can be easily detected by a piezoelectric biosensor. This type of assay was described for the diagnosis of various pathological states with quartz crystal microbalance as the sensor platform [94,95,96,97]. This type of biosensor remains rare for development of new point-of-care tests for the diagnosis, but they are quite convenient for the purpose and interest for piezoelectric platform can grow in the future. The major advantage of the piezoelectric platform is the possibility of making a label-free assay specifically measuring mass of the analyte catch on the biosensor surface by determining the oscillation frequency dropping due to the bound mass. The principle of antibody assays by a piezoelectric biosensor can be learned from Figure 3. An ultrasonic guided wave sensor using lithium niobate piezoelectric wafer was prepared and verified by Mandal and coworkers [98]. The piezoelectric biosensor contained the S protein immobilized through gold nanoparticles and exerted the signal when a sample containing total anti-SARS-CoV-2 antibodies presented. The authors did not report analytical specifications, such as the limit of detection or sensitivity of their method, but introduced their assay as a platform for the next development. 

Biosensing platforms can serve to precise determine the efficacy of vaccination efficacy. Such an approach was examined, for instance, in the study by Kim and collaborators, where a label-free surface-enhanced Raman scattering biosensing platform containing metallic nanostructures was developed to prove anti-SARS-CoV-2 antibodies in tears [99]. The non-linear optical assay was tested on volunteers vaccinated with Oxford-Astra Zeneca AZD1222 vaccine. The assay was able to detect IgG with a detection limit of 10^−14^ mol/L with good reproducibility of the results, as the relative standard deviation was under 3%. The high sensitivity was necessary to allow the use of tear fluid samples where the number of antibodies is quite low compared to blood. The fact that the assay can be used for the analysis of samples where noninvasive collection is possible represents a substantial advantage. The aforementioned biosensors are summarized in Table 1.

## 5. Biosensors for Measuring SARS-CoV-2 and Its Determining Parts

Biosensors for the direct detection of SARS-CoV-2 under point-of-care conditions can represent a wide group of analytical devices using immunochemical or molecular biology approaches to identify the unique determinants distinguishing SARS-CoV-2 from other viruses or biological materials. In addition to biosensors for use in point-of-care conditions, there are also highly sophisticated biosensors suitable for SARS-CoV-2 detection [100,101,102,103,104,105]. In this review, portable platforms ready to be used in the conditions of home care or other outside laboratory environments are taken into account. An overview of the biosensors discussed is given in Table 2. 

A biosensor that works on the principle of electrochemiluminescence was developed to detect very low concentrations of SARS-CoV-2 DNA [106]. The biosensor used tris(bipyridine)ruthenium (II) chloride as the luminophore in combination with carbon nanodots as the reactant. The surface of the biosensor was covered with gold nanotriangles and gold nanoparticles with SARS-CoV-2-specific single-stranded oligonucleotide hybridization with SARS-CoV-2 DNA in a tested sample. The assay proved to be very sensitive as the limit of detection for oligonucleotides from SARS-CoV-2 was equal to 514 amol/L and the assay was resistant to interference by oligonucleotides from the influenza virus at the same time. 

A piezoelectric biosensor based on a quartz crystal microbalance resonator was developed for a label-free detection of SARS-CoV-2 virions [107]. The biosensor contained an immobilized antibody specific to the N protein of SARS-CoV-2 and was suitable for a label-free assay with a limit of detection of 6.7 × 10^3^ PFU/mL and was successfully validated on nasopharyngeal swab samples previously analyzed by reverse transcriptase PCR. In another experiment, an electrochemical biosensor working on the principle of potentiometry and based on a dual gate field-effect transistor was developed as a portable analytical device [108]. While one gate of the biosensor contained immobilized angiotensin converting enzyme 2 (ACE2), the second gate had an immobilized antibody specific to the S protein of SARS-CoV-2. Surrogate viral particles prepared from liposomes covered with avidin and S protein served as an analyte during biosensor testing. Both gates of the biosensor had the same limit of detection equal to approximately 165 surrogated viral particles per milliliter of a sample for an assay lasting 20 min. The dual assay based on both ACE2 and an antibody as biorecognition elements of the biosensor was an original approach because an assay based on the recognition ability of an antibody can be highly specific, but there is a risk of false negativity when a mutant SARS-CoV-2 is detected. On the other hand, the interaction between ACE2 and SARS-CoV-2 will be a sensitive one but false positivity could occur. Combining information from the two gates can provide highly plausible confirmation or the neglect of SARS-CoV-2 presence in a tested sample.

**Figure 4:**
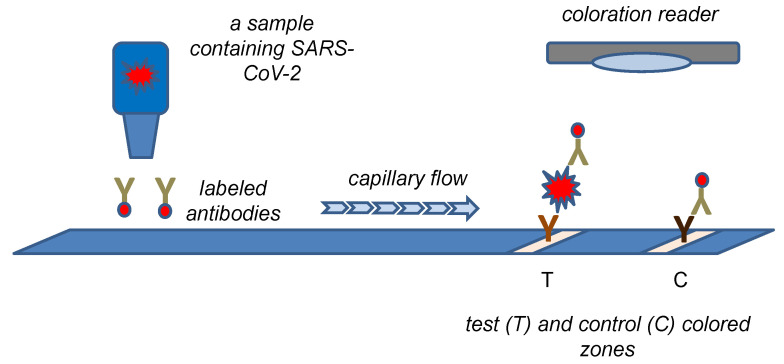
A biosensor based on the principle of the lateral flow test with coloration reader to measure color density.

The principle of a standard lateral flow test with a color density can be easily adopted for a biosensor construction. Such an approach combines reliability of the lateral flow tests with semi-quantification of an analyte content. The common principle of such test is depicted in Figure 4. Lee et al. constructed a colorimetric biosensor on the platform of lateral flow tests using monoclonal antibodies against N protein and colored cellulose nanobeads as a label attached to the secondary antibody [109]. The assay was organized as a semiquantitative one with a portable line analyzer as the measuring device. The limit of detection of 100 pg of N protein and 1400 TCID_50_ (median tissue culture infectious dose) in a tested sample was calculated for an assay lasting 15 min. A similar concept based on the lateral flow test was chosen in the study by Kim et al. [110]. The researchers prepared fragmented anti-N protein antibodies and also used colored cellulose nanobeads. When the assay was combined with the portable line analyzer as a measuring device, a limit of detection of 2 ng for N protein or 2.5 × 10^4^ PFU was achieved. 

The recognition capability of antibodies was also used in a biosensor manufactured by Kim and coworkers [111]. They chose quasi-free-standing bilayer epitaxial graphene on silicon carbide with immobilized anti S1 protein antibody as recognition element and titanium–gold electrodes. The electrochemical assay exerted a limit of detection of 60 SARS-CoV-2 copies per milliliter of sample, and a limit of detection of 1 ag/mL for the pure S1 protein was achieved.

Another concept for manufacturing a simple biosensor suitable for mass production and point-of-care conditions was proposed by Yakoh et al. [112]. A folding paper with integrated ink-printed working, counter and reference electrodes was the assay platform. The electrochemical biosensor was prepared in two variants applicable for the test of antibodies against SARS-CoV-2 and the direct detection of SARS-CoV-2 by recognizing its S protein. The working electrode of the biosensor contained graphene oxide with immobilized S protein for detection of antibodies in blood samples or anti S protein IgM when SARS-CoV-2 was detected. The redox system of ferrocyanide—ferricyanide in combination with square wave voltammetry served for the assay. Access to the redox reaction to the electrode was restricted when an analyte became bound. The limit of detection was equal to 0.96 ng/mL for IgG and 0.14 for IgM when the diagnosis of COVID-19 was made by recognition of specific antibodies against the S protein. The S protein was assayed with the limit of detection of 0.11 ng/mL. 

## 6. Conclusions

Biosensors are promising analytical devices suitable for various outdoor applications and field and point-of-care tests. The reviewed applications for COVID-19 diagnosis by detecting SARS-CoV-2 virus parts or recognizing specific anti- SARS-CoV-2 antibodies exert great potential to be implemented and used in medical praxis. Although further development of technical instruments controlling the biosensors and solving of industrial feasibility should be performed, the primary research on the biosensors for COVID-19 diagnosis resulted in the promising proposals. Further research on the biosensor would simplify the manufacture of devices with fewer costs and lower requirements for users. Current research made biosensors fully competitive with other analytical and diagnostic point-of-care tests, and biosensors can help to improve the care of patients suffering with COVID-19 and make the diagnosis of the disease more accurate and available and the results of the assay more plausible. When compared to the two types of biosensors for COVID-19 diagnosis, the biosensors for the anti-SARS-CoV-2 antibodies assay seems to be less practical for point-of-care conditions because of potential problems with blood sample collection by inexperienced staff and possible false negative diagnosis in the early phases of the disease. Nevertheless, both types of the biosensors have potential to be used in medical praxis. 

The practical implementation of findings and new technologies in the field of biosensors for the diagnosis of COVID-19 can be expected and commercialization of the inventions will be one of the next steps. The scale of implementation of the findings will depend on the further development of the COVID-19 pandemic and the necessity to solve it. However, findings related to COVID-19 diagnosis can be easily adapted to the diagnosis of other infectious diseases and so they can gain practical impact even in the case of successful suppression of the COVID-19 pandemic. 

## Figures and Tables

**Table 1:** Anti-COVID-19 point-of-care biosensors for measuring anti-SARS-CoV-2 antibodies.

Device	Type of Technique	Type of Detected Antibodies	Specifications	References
multiplexed grating-coupled fluorescent plasmonics biosensor with immobilized S and N proteins	spectroscopic technique	anti S and N proteins Ig	selectivity 100% and sensitivity 87%	[84]
surface plasmon resonance biosensor containing S protein	spectroscopic technique	IgG, IgM, and IgA specific to receptor binding domain	sensitivity was equal to 93% and specificity to 100%	[85]
nanoplasmonic biosensing platform with immobilized S protein on gold triangular nanoprisms	spectroscopic technique	IgG specific to S protein	limit of detection equal 30 amol/L, specificity 90%, sensitivity 100%	[86]
colorimetric vertical-flow immunoassay biosensor containing gold nanoparticles labeled antibodies	spectroscopic technique	IgG and IgM specific to S protein	assay time 2 min, applicable for interstitial fluid as a sample	[87]
voltammetric biosensor with receptor binding domain bioconjugate with SARS-CoV-2 antigen	electrochemical technique	IgG against receptor binding domain	limit of detection 1.0 pg/mL, sample size 40 µL, assay time 15 min	[88]
optofluidic fluorescence biosensor containing S protein	spectroscopic technique	IgG specific against S protein	limit of detection 12.5 ng/mL, sample sized 100 µL, assay lasting 25 min	[89]
Fresnel reflection microfluidic biosensor	spectroscopic technique	IgG and IgM specific to S protein	limit of detection 0.82 ng/mL for IgM and 0.45 ng/mL for IgG	[90]
field effect transistor voltammetric recording formation of an immunocomplex	electrochemical technique	IgG specific to S protein	limit of detection 1 pg/mL	[91]
avidity testing-on-a-probe biosensor	spectroscopic technique	total antibodies specific to S protein	approximate limit of detection 2.5 µg/mL of IgG or IgM	[92]
piezoelectric biosensor	piezoelectrical technique	total antibodies specific to S protein	N/A	[98]
surface-enhanced Raman scattering biosensing platform	spectroscopic technique	IgG created after the AZD1222 vaccine application	limit of detection 10^−14^ mol/L, the relative standard deviation was under 3%	[99]

**Table 2:** Point-of-care biosensors for SARS-CoV-2 assay.

Device	Type of Technique	Detected Part of SARS-CoV-2	Specifications	References
electrochemical biosensor with oligonucleotide immobilized on gold nanostructures	electrochemical technique	specific oligonucleotide	limit of detection 514 amol/L	[106]
quartz crystal microbalance biosensor with bound antibody	piezoelectrical technique	N protein	limit of detection 6.7 × 10^3^ PFU/mL	[107]
potentiometric biosensor with dual gate field-effect transistor, immobilized ACE2, and an antibody	electrochemical technique	S protein via interaction with ACE2 or an antibody	limit of detection 165 viral particles/mL, assay time 20 min	[108]
lateral flow test with antibodies labeled by colored cellulose nanobeads and line analyzer	spectroscopic technique	N protein	limit of detection 100 pg of N protein respectively 1400 TCID_50_, assay time 15 min	[109]
lateral flow test with fragmented antibodies labeled by colored cellulose nanobeads and line analyzer	spectroscopic technique	N protein	limit of detection 2 ng for N protein or 2.5 × 10^4^ PFU	[110]
electrochemical biosensor with graphene on silicon carbide and immobilized anti S1 protein antibody	electrochemical technique	S1 protein	limit of detection 60 virus copies/mL or 1 ag/mL for pure S1 protein	[111]
paper electrochemical biosensor containing either anti S protein antibody (or S protein)	electrochemical technique	S protein and possible assay of anti S protein antibodies	limit of detection 0.96 ng/mL for IgG, 0.14 for IgM, 0.11 ng/mL for S protein	[112]

## Data Availability

Not applicable.

## References

[1] Eibensteiner F., Ritschl V., Stamm T., Cetin A., Schmitt C.P., Ariceta G., Bakkaloglu S., Jankauskiene A., Klaus G., Paglialonga F. (2021). Countermeasures against COVID-19: How to navigate medical practice through a nascent, evolving evidence base—A European multicentre mixed methods study. BMJ Open.

[2] Kong L.C., Hu Y., Wang Q., Chen X.D., Yao T., Wang Y., Jin H., Fan L.J., Du W. (2021). Could COVID-19 pandemic be stopped with joint efforts of travel restrictions and public health countermeasures? A modelling study. BMJ Open.

[3] Vichiensan V., Hayashi Y., Kamnerdsap S. (2021). COVID-19 Countermeasures and Passengers’ Confidence of Urban Rail Travel in Bangkok. Sustainability.

[4] Si R.S., Yao Y.M., Zhang X.Q., Lu Q., Aziz N. (2021). Investigating the Links Between Vaccination Against COVID-19 and Public Attitudes Toward Protective Countermeasures: Implications for Public Health. Front. Public Health.

[5] Zhu H., Lu H.Z. (2020). The development of a quarantine strategy is an important path to a normalized response to COVID-19. BioSci. Trends.

[6] Traylor A.M., Tannenbaum S.I., Thomas E.J., Salas E. (2021). Helping Healthcare Teams Save Lives During COVID-19: Insights and Countermeasures From Team Science. Am. Psychol..

[7] Mohsin A.K.M., Lei H.Z., Hossain S.F.A. (2021). Impact of COVID-19 Pandemic on Consumer Economy: Countermeasures Analysis. Sage Open.

[8] Wang L.J., Li C.D., Chen X.H., Zhu L.L. (2020). Causal Relationship Between the Spread of the COVID-19 and Geopolitical Risks in Emerging Economies. Front. Public Health.

[9] Butt A.S. (2022). Supply chains and COVID-19: Impacts, countermeasures and post-COVID-19 era. Int. J. Logist. Manag..

[10] Liu Y.W., Cui Q., Liu Y., Zhang J.Z., Zhou M.F., Ali T., Yang L.Y., Feng K.S., Hubacek K., Li X.B. (2021). Countermeasures against economic crisis from COVID-19 pandemic in China: An analysis of effectiveness and trade-offs. Struct. Chang. Econ. Dyn..

[11] Li Y., Wang X.J., Wang W. (2021). The Impact of COVID-19 on Cancer. Infect. Drug Resist..

[12] Zhao Z., Huang C.F., Huang Z.Y., Lin F.J., He Q.L., Tao D., Jaffrezic-Renault N., Guo Z.Z. (2021). Advancements in electrochemical biosensing for respiratory virus detection: A review. Trac-Trends Anal. Chem..

[13] Tran V.V., Tran N.H.T., Hwang H.S., Chang M. (2021). Development strategies of conducting polymer-based electrochemical biosensors for virus biomarkers: Potential for rapid COVID-19 detection. Biosens. Bioelectron..

[14] Choi J.R. (2020). Development of Point-of-Care Biosensors for COVID-19. Front. Chem..

[15] Yue J., Xu H., Zhou Y., Liu W., Han X.F., Mao Q., Li S.X., Tam L.S., Ma J., Liu W. (2021). Dyslipidemia Is Related to Mortality in Critical Patients With Coronavirus Disease 2019: A Retrospective Study. Front. Endocrinol..

[16] Gonzalez-Garcia N., Miranda-Lora A.L., Garduno-Espinosa J., Granados-Riveron J.T., Mendez-Galvan J.F., Nieto-Zermeno J., Castilla-Peon M.F. (2021). International heterogeneity in coronavirus disease 2019 pediatric mortality rates. Bol. Med. Hosp. Infant. Mex..

[17] Hernandez-Romieu A.C., Adelman M.W., Hockstein M.A., Robichaux C.J., Edwards J.A., Fazio J.C., Blum J.M., Jabaley C.S., Caridi-Scheible M., Martin G.S. (2020). Timing of Intubation and Mortality Among Critically Ill Coronavirus Disease 2019 Patients: A Single-Center Cohort Study. Crit. Care Med..

[18] Barioni E.M.S., Nascimento C., Amaral T.L.M., Ramalho Neto J.M., Prado P.R.D. (2022). Clinical indicators, nursing diagnoses, and mortality risk in critically ill patients with COVID-19: A retrospective cohort. Rev. Esc. Enferm. USP.

[19] Cui D., Wang Y.M., Huang L.X., Gu X.Y., Huang Z.S., Mu S.R., Wang C., Cao B. (2022). Rheumatic Symptoms Following Coronavirus Disease 2019 (COVID-19): A Chronic Post-COVID-19 Condition. Open Forum Infect. Dis..

[20] Kadirvelu B., Burcea G., Quint J.K., Costelloe C.E., Faisal A.A. (2022). Variation in global COVID-19 symptoms by geography and by chronic disease: A global survey using the COVID-19 Symptom Mapper. EClinicalMedicine.

[21] Galal I., Hussein A., Amin M.T., Saad M.M., Zayan H.E.E., Abdelsayed M.Z., Moustafa M.M., Ezzat A.R., Helmy R.E.D., Abd Elaal H.K. (2021). Determinants of persistent post-COVID-19 symptoms: Value of a novel COVID-19 symptom score. Egypt. J. Bronchol..

[22] Luo Y.M., Wu J., Lu J.Y., Xu X., Long W., Yan G.J., Tang M.Y., Zou L., Xu D.Z., Zhuo P. (2020). Investigation of COVID-19-related symptoms based on factor analysis. Ann. Pallliat. Med..

[23] Fernandez-de-las-Penas C., Martin-Guerrero J.D., Pellicer-Valero O.J., Navarro-Pardo E., Gomez-Mayordomo V., Cuadrado M.L., Arias-Navalon J.A., Cigaran-Mendez M., Hernandez-Barrera V., Arendt-Nielsen L. (2022). Female Sex Is a Risk Factor Associated with Long-Term Post-COVID Related-Symptoms but Not with COVID-19 Symptoms: The LONG-COVID-EXP-CM Multicenter Study. J. Clin. Med..

[24] Soma P., Bester J. (2022). Pathophysiological Changes in Erythrocytes Contributing to Complications of Inflammation and Coagulation in COVID-19. Front. Physiol..

[25] Lamb L.E., Dhar N., Timar R., Wills M., Dhar S., Chancellor M.B. (2020). COVID-19 inflammation results in urine cytokine elevation and causes COVID-19 associated cystitis (CAC). Med. Hypotheses.

[26] Wong R.S.Y. (2021). Inflammation in COVID-19: From pathogenesis to treatment. Int. J. Clin. Exp. Pathol..

[27] Tan Q.Q., He L.J., Meng X.J., Wang W., Pan H.D., Yin W.G., Zhu T.C.A., Huang X., Shan H. (2021). Macrophage biomimetic nanocarriers for anti-inflammation and targeted antiviral treatment in COVID-19. J. Nanobiotechnol..

[28] Mester A., Benedek I., Rat N., Tolescu C., Polexa S.A., Benedek T. (2021). Imaging Cardiovascular Inflammation in the COVID-19 Era. Diagnostics.

[29] Johnson J.N., Loriaux D.B., Jenista E., Kim H.W., Baritussio A., De Garate Iparraguirre E., Bucciarelli-Ducci C., Denny V., O’Connor B., Siddiqui S. (2022). Society for Cardiovascular Magnetic Resonance 2021 cases of SCMR and COVID-19 case collection series. J. Cardiovasc. Magn. Reson..

[30] Shim S.R., Kim S.J., Hong M., Lee J., Kang M.G., Han H.W. (2022). Diagnostic Performance of Antigen Rapid Diagnostic Tests, Chest Computed Tomography, and Lung Point-of-Care-Ultrasonography for SARS-CoV-2 Compared with RT-PCR Testing: A Systematic Review and Network Meta-Analysis. Diagnostics.

[31] Barbas C.S.V. (2022). Thoracic Computed Tomography to Assess ARDS and COVID-19 Lungs. Front. Physiol..

[32] Jenkins H.H., Lopez A.A.T., Tarantini F.S., Tomlin H., Scales D., Lee I.N., Wu S., Hyde R., Lis-Slimak K., Byaruhanga T. (2022). Performance evaluation of a non-invasive one-step multiplex RT-qPCR assay for detection of SARS-CoV-2 direct from saliva. Sci. Rep..

[33] Mannan N., Raihan R., Parvin U.S., Fazle Akbar S.M., Reza M.S., Islam S., Kundu J., Noman A.A., Fakhruddin M., Billaha M. (2022). Detection of SARS-CoV-2 RNA by Reverse Transcription-Polymerase Chain Reaction (RT-PCR) on Self-Collected Nasal Swab Compared With Professionally Collected Nasopharyngeal Swab. Cureus.

[34] Ali H., Alkhaursi K., Holton T. (2022). Development of a colorimetric RT-LAMP assay for the detection of SARS-COV-2 isolated from Oman. J. Infect. Dev. Ctries..

[35] Khan S.H., Zaidi S.K., Gilani M. (2022). PCR to CRISPR: Role of Nucleic Acid Tests (NAT) in detection of COVID-19. J. Pak. Med. Assoc..

[36] Yu Z., Xu L., Lyu W., Shen F. (2022). Parallel multistep digital analysis SlipChip demonstrated with the quantification of nucleic acid by digital LAMP-CRISPR. Lab Chip.

[37] Kashir J., Yaqinuddin A. (2020). Loop mediated isothermal amplification (LAMP) assays as a rapid diagnostic for COVID-19. Med. Hypotheses.

[38] Augustine R., Hasan A., Das S., Ahmed R., Mori Y., Notomi T., Kevadiya B.D., Thakor A.S. (2020). Loop-Mediated Isothermal Amplification (LAMP): A Rapid, Sensitive, Specific, and Cost-Effective Point-of-Care Test for Coronaviruses in the Context of COVID-19 Pandemic. Biology.

[39] Balck A., Föh B., Borsche M., Rahmöller J., Vollstedt E.J., Waldeck F., Käding N., Twesten C., Mischnik A., Gillessen-Kaesbach G. (2022). Protocol of the Luebeck longitudinal investigation of SARS-CoV-2 infection (ELISA) study—A prospective population-based cohort study. BMC Public Health.

[40] Ramos A., Araújo B., Lacerda L., Flora A.L., Ribeiro L., Patrício E., Cardoso M.J., Guimarães J.T. (2022). SARS-CoV-2 seroprevalence in healthcare workers: The experience of a Portuguese COVID-19 front-line hospital during the 1st pandemic wave. Porto. Biomed. J..

[41] Donoso Mantke O., Corman V.M., Taddei F., McCulloch E., Niemeyer D., Grumiro L., Dirani G., Wallace P.S., Drosten C., Sambri V. (2022). Importance of external quality assessment for SARS-CoV-2 antigen detection during the COVID-19 pandemic. J. Clin. Virol..

[42] Schwarze M., Krizsan A., Brakel A., Pohl F., Volke D., Hoffmann R. (2022). Cross-Reactivity of IgG Antibodies and Virus Neutralization in mRNA-Vaccinated People Against Wild-Type SARS-CoV-2 and the Five Most Common SARS-CoV-2 Variants of Concern. Front. Immunol..

[43] Sakyi A., Laing E., Ephraim R., Asibey O., Sadique O. (2015). Evaluation of analytical errors in a clinical chemistry laboratory: A 3 year experience. Ann. Med. Health Sci. Res..

[44] Al-Ghaithi H., Pathare A., Al-Mamari S., Villacrucis R., Fawaz N., Alkindi S. (2017). Impact of Educational Activities in Reducing Pre-Analytical Laboratory Errors: A quality initiative. Sultan Qaboos Univ. Med. J..

[45] Sturgeon C.M. (2013). External quality assessment of hormone determinations. Best Pract. Res. Clin. Endocrinol. Metab..

[46] Lee G.R., Fitzgibbon M.C., O’Shea P. (2016). Laboratory services: Regaining and maintaining control. Int. J. Health Care Qual. Assur..

[47] Wallace P.S., MacKay W.G. (2013). Quality in the molecular microbiology laboratory. Methods Mol. Biol..

[48] Somborac Bačura A., Dorotić M., Grošić L., Džimbeg M., Dodig S. (2021). Current status of the lateral flow immunoassay for the detection of SARS-CoV-2 in nasopharyngeal swabs. Biochem. Med..

[49] Zhou Y., Wu Y., Ding L., Huang X., Xiong Y. (2021). Point-of-care COVID-19 diagnostics powered by lateral flow assay. Trends Analyt. Chem..

[50] Wang J.J., Zhang N., Richardson S.A., Wu J.V. (2021). Rapid lateral flow tests for the detection of SARS-CoV-2 neutralizing antibodies. Expert. Rev. Mol. Diagn..

[51] Pohanka M. (2021). Point-of-Care Diagnoses and Assays Based on Lateral Flow Test. Int. J. Anal. Chem..

[52] Choi S., Choi E.Y., Kim D.J., Kim J.H., Kim T.S., Oh S.W. (2004). A rapid, simple measurement of human albumin in whole blood using a fluorescence immunoassay (I). Clin. Chim. Acta.

[53] Wu Y.H., Zhou Y.F., Leng Y.K., Lai W.H., Huang X.L., Xiong Y.H. (2020). Emerging design strategies for constructing multiplex lateral flow test strip sensors. Biosens. Bioelectron..

[54] Ngom B., Guo Y.C., Wang X.L., Bi D.R. (2010). Development and application of lateral flow test strip technology for detection of infectious agents and chemical contaminants: A review. Anal. Bioanal. Chem..

[55] Machiesky L., Cote O., Kirkegaard L.H., Mefferd S.C., Larkin C. (2019). A rapid lateral flow immunoassay for identity testing of biotherapeutics. J. Immunol. Methods.

[56] Hendrickson O.D., Byzova N.A., Zvereva E.A., Zherdev A.V., Dzantiev B.B. (2021). Sensitive lateral flow immunoassay of an antibiotic neomycin in foodstuffs. J. Food Sci. Technol..

[57] Beloglazova N.V., Shmelin P.S., Eremin S.A. (2016). Sensitive immunochemical approaches for quantitative (FPIA) and qualitative (lateral flow tests) determination of gentamicin in milk. Talanta.

[58] Dou L.N., Zhao B.X., Bu T., Zhang W.T., Huang Q., Yan L.Z., Huang L.J., Wang Y.R., Wang J.L., Zhang D.H. (2018). Highly sensitive detection of a small molecule by a paired labels recognition system based lateral flow assay. Anal. Bioanal. Chem..

[59] Alnajrani M.N., Alsager O.A. (2019). Lateral flow aptasensor for progesterone: Competitive target recognition and displacement of short complementary sequences. Anal. Biochem..

[60] Yang H.L., Wang Y.R., Liu S.Y., Ouyang H., Lu S.G., Li H.T., Fu Z.F. (2021). Lateral flow assay of methicillin-resistant Staphylococcus aureus using bacteriophage cellular wall-binding domain as recognition agent. Biosens. Bioelectron..

[61] Lee K.W., Yu Y.C., Chun H.J., Jang Y.H., Han Y.D., Yoon H.C. (2020). Instrumentation-Free Semiquantitative Immunoanalysis Using a Specially Patterned Lateral Flow Assay Device. Biosensors.

[62] Soleimani R., Deckers C., Huang T.D., Bogaerts P., Evrard S., Wallemme I., Habib B., Rouze P., Denis O. (2021). Rapid COVID-19 antigenic tests: Usefulness of a modified method for diagnosis. J. Med. Virol..

[63] Frnda J., Durica M. (2021). On Pilot Massive COVID-19 Testing by Antigen Tests in Europe. Case Study: Slovakia. Infect. Dis. Rep..

[64] Candel F.J., Barreiro P., San Roman J., Abanades J.C., Barba R., Barberan J., Bibiano C., Canora J., Canton R., Calvo C. (2020). Recommendations for use of antigenic tests in the diagnosis of acute SARS-CoV-2 infection in the second pandemic wave: Attitude in different clinical settings. Rev. Esp. Quim..

[65] Scohy A., Anantharajah A., Bodeus M., Kabamba-Mukadi B., Verroken A., Rodriguez-Villalobos H. (2020). Low performance of rapid antigen detection test as frontline testing for COVID-19 diagnosis. J. Clin. Virol..

[66] Yamayoshi S., Sakai-Tagawa Y., Koga M., Akasaka O., Nakachi I., Koh H., Maeda K., Adachi E., Saito M., Nagai H. (2020). Comparison of Rapid Antigen Tests for COVID-19. Viruses.

[67] Kyosei Y., Yamura S., Namba M., Yoshimura T., Watabe S., Ito E. (2021). Antigen tests for COVID-19. Biophys. Physicobiol..

[68] Siddiqui Z.K., Chaudhary M., Robinson M.L., McCall A.B., Peralta R., Esteve R., Callahan C.W., Manabe Y.C., Campbell J.D., Johnson J.K. (2021). Implementation and Accuracy of BinaxNOW Rapid Antigen COVID-19 Test in Asymptomatic and Symptomatic Populations in a High-Volume Self-Referred Testing Site. Microbiol. Spectr..

[69] Fearon E., Buchan I.E., Das R., Davis E.L., Fyles M., Hall I., Hollingsworth T.D., House T., Jay C., Medley G.F. (2021). SARS-CoV-2 antigen testing: Weighing the false positives against the costs of failing to control transmission. Lancet Respir. Med..

[70] Shey M.S., Schmidt B.M., Wiysonge C.S. (2020). Antibody tests for diagnosing COVID-19: How relevant are they?. Pan Afr. Med. J..

[71] Zhao J., Yuan Q., Wang H., Liu W., Liao X., Su Y., Wang X., Yuan J., Li T., Li J. (2020). Antibody Responses to SARS-CoV-2 in Patients With Novel Coronavirus Disease 2019. Clin. Infect. Dis..

[72] Ong D.S.Y., Fragkou P.C., Schweitzer V.A., Chemaly R.F., Moschopoulos C.D., Skevaki C. (2021). How to interpret and use COVID-19 serology and immunology tests. Clin. Microbiol. Infect..

[73] Zollner A., Watschinger C., Rössler A., Farcet M.R., Penner A., Böhm V., Kiechl S.J., Stampfel G., Hintenberger R., Tilg H. (2021). B and T cell response to SARS-CoV-2 vaccination in health care professionals with and without previous COVID-19. EBioMedicine.

[74] Zurac S., Nichita L., Mateescu B., Mogodici C., Bastian A., Popp C., Cioplea M., Socoliu C., Constantin C., Neagu M. (2021). COVID-19 vaccination and IgG and IgA antibody dynamics in healthcare workers. Mol. Med. Rep..

[75] Schrezenmeier E., Bergfeld L., Hillus D., Lippert J.D., Weber U., Tober-Lau P., Landgraf I., Schwarz T., Kappert K., Stefanski A.L. (2021). Immunogenicity of COVID-19 Tozinameran Vaccination in Patients on Chronic Dialysis. Front. Immunol..

[76] Wang J., Hou Z., Liu J., Gu Y., Wu Y., Chen Z., Ji J., Diao S., Qiu Y., Zou S. (2021). Safety and immunogenicity of COVID-19 vaccination in patients with non-alcoholic fatty liver disease (CHESS2101): A multicenter study. J. Hepatol..

[77] Tretyn A., Szczepanek J., Skorupa M., Jarkiewicz-Tretyn J., Sandomierz D., Dejewska J., Ciechanowska K., Jarkiewicz-Tretyn A., Koper W., Pałgan K. (2021). Differences in the Concentration of Anti-SARS-CoV-2 IgG Antibodies Post-COVID-19 Recovery or Post-Vaccination. Cells.

[78] Kocagoz T., Can O., Yurttutan Uyar N., Aksoy E., Polat T., Cankaya D., Karakus B., Mozioglu E., Kocagoz S. (2021). Simple concentration method enables the use of gargle and mouthwash instead of nasopharyngeal swab sampling for the diagnosis of COVID-19 by PCR. Eur. J. Clin. Microbiol. Infect. Dis..

[79] Pohanka M. (2021). Point-of-care diagnosis of COVID-19 disease based on antigen tests. Bratisl. Med. J..

[80] Pohanka M. (2021). COVID-19 molecular level laboratory diagnoses. Bratisl. Med. J..

[81] Pandolfi L., Fossali T., Frangipane V., Bozzini S., Morosini M., D’Amato M., Lettieri S., Urtis M., Di Toro A., Saracino L. (2020). Broncho-alveolar inflammation in COVID-19 patients: A correlation with clinical outcome. BMC Pulm. Med..

[82] Zhang Y., Ong C.M., Yun C., Mo W., Whitman J.D., Lynch K.L., Wu A.H.B. (2021). Diagnostic Value of Nucleocapsid Protein in Blood for SARS-CoV-2 Infection. Clin. Chem..

[83] Thudium R.F., Stoico M.P., Høgdall E., Høgh J., Krarup H.B., Larsen M.A.H., Madsen P.H., Nielsen S.D., Ostrowski S.R., Palombini A. (2021). Early Laboratory Diagnosis of COVID-19 by Antigen Detection in Blood Samples of the SARS-CoV-2 Nucleocapsid Protein. J. Clin. Microbiol..

[84] Cady N.C., Tokranova N., Minor A., Nikvand N., Strle K., Lee W.T., Page W., Guignon E., Pilar A., Gibson G.N. (2021). Multiplexed detection and quantification of human antibody response to COVID-19 infection using a plasmon enhanced biosensor platform. Biosens. Bioelectron..

[85] Schasfoort R.B.M., van Weperen J., van Amsterdam M., Parisot J., Hendriks J., Koerselman M., Karperien M., Mentink A., Bennink M., Krabbe H. (2021). High throughput surface plasmon resonance imaging method for clinical detection of presence and strength of binding of IgM, IgG and IgA antibodies against SARS-CoV-2 during CoViD-19 infection. MethodsX.

[86] Masterson A.N., Sardar R. (2022). Selective Detection and Ultrasensitive Quantification of SARS-CoV-2 IgG Antibodies in Clinical Plasma Samples Using Epitope-Modified Nanoplasmonic Biosensing Platforms. ACS Appl. Mater. Interfaces.

[87] Bao L.L., Park J., Shim S., Yoneda M., Kai C., Kim B., Ieee A rapid COVID-19 diagnostic device integrating porous microneedles and the paper-based immunoassay biosensor. Proceedings of the 10th IEEE CPMT Symposium Japan (ICSJ).

[88] Mattioli I.A., Castro K.R., Macedo L.J.A., Sedenho G.C., Oliveira M.N., Todeschini I., Vitale P.M., Ferreira S.C., Manuli E.R., Pereira G.M. (2022). Graphene-based hybrid electrical-electrochemical point-of-care device for serologic COVID-19 diagnosis. Biosens. Bioelectron..

[89] Song D., Liu J.Y., Xu W.J., Han X.Z., Wang H.L., Cheng Y., Zhuo Y.X., Long F. (2021). Rapid and quantitative detection of SARS-CoV-2 IgG antibody in serum using optofluidic point-of-care testing fluorescence biosensor. Talanta.

[90] Xu W.J., Liu J.Y., Song D., Li C.S., Zhu A.N., Long F. (2021). Rapid, label-free, and sensitive point-of-care testing of anti-SARS-CoV-2 IgM/IgG using all-fiber Fresnel reflection microfluidic biosensor. Microchim. Acta.

[91] Chen M.R., Cui D.Z., Zhao Z.Y., Kang D., Li Z., Albawardi S., Alsageer S., Alamri F., Alhazmi A., Amer M.R. (2022). Highly sensitive, scalable, and rapid SARS-CoV-2 biosensor based on In_2_O_3_ nanoribbon transistors and phosphatase. Nano Res..

[92] Yang H.S., Racine-Brzostek S.E., Karbaschi M., Yee J., Dillard A., Steel P.A.D., Lee W.T., McDonough K.A., Qiu Y., Ketas T.J. (2021). Testing-on-a-probe biosensors reveal association of early SARS-CoV-2 total antibodies and surrogate neutralizing antibodies with mortality in COVID-19 patients. Biosens. Bioelectron.

[93] Racine-Brzostek S.E., Karbaschi M., Gaebler C., Klasse P.J., Yee J., Caskey M., Yang H.S., Hao Y., Sukhu A., Rand S. (2021). TOP-Plus Is a Versatile Biosensor Platform for Monitoring SARS-CoV-2 Antibody Durability. Clin. Chem..

[94] Pohanka M. (2018). Piezoelectric Immunosensor for the Determination of Immunoglobulin G. Int. J. Electrochem. Sc..

[95] Li H., Long M., Su H.Y., Tan L., Shi X.W., Du Y.M., Luo Y., Deng H.B. (2022). Carboxymethyl chitosan assembled piezoelectric biosensor for rapid and label-free quantification of immunoglobulin Y. Carbohydr. Polym..

[96] Zhou L.J., Kato F., Ogi H. (2021). Sensitive label-free immunoglobulin G detection using a MEMS quartz crystal microbalance biosensor with a 125 MHz wireless quartz resonator. Jpn. J. Appl. Phys..

[97] Liu Y., Yu X., Zhao R., Shangguan D.H., Bo Z.Y., Liu G.Q. (2003). Real time kinetic analysis of the interaction between immunoglobulin G and histidine using quartz crystal microbalance biosensor in solution. Biosens. Bioelectron..

[98] Mandal D., Indaleeb M.M., Younan A., Banerjee S. (2022). Piezoelectric point-of-care biosensor for the detection of SARS-COV-2 (COVID-19) antibodies. Sens. Bio-Sens. Res..

[99] Kim W., Kim S., Han J., Kim T.G., Bang A., Choi H.W., Min G.E., Shin J.H., Moon S.W., Choi S. (2022). An excitation wavelength-optimized, stable SERS biosensing nanoplatform for analyzing adenoviral and AstraZeneca COVID-19 vaccination efficacy status using tear samples of vaccinated individuals. Biosens. Bioelectron..

[100] Wu Q., Wu W., Chen F.F., Ren P. (2022). Highly sensitive and selective surface plasmon resonance biosensor for the detection of SARS-CoV-2 spike S1 protein. Analyst.

[101] Kumar A., Kumar A., Srivastava S.K. (2022). Silicon Nitride-BP-Based Surface Plasmon Resonance Highly Sensitive Biosensor for Virus SARS-CoV-2 Detection. Plasmonics.

[102] Saad Y., Gazzah M.H., Mougin K., Selmi M., Belmabrouk H. (2022). Sensitive Detection of SARS-CoV-2 Using a Novel Plasmonic Fiber Optic Biosensor Design. Plasmonics.

[103] Cennamo N., Pasquardini L., Arcadio F., Lunelli L., Vanzetti L., Carafa V., Altucci L., Zeni L.G. (2021). SARS-CoV-2 spike protein detection through a plasmonic D-shaped plastic optical fiber aptasensor. Talanta.

[104] Zheng Y.Q., Bian S.M., Sun J.C., Wen L.Y., Rong G.G., Sawan M. (2022). Label-Free LSPR-Vertical Microcavity Biosensor for On-Site SARS-CoV-2 Detection. Biosensors.

[105] Peng Y., Pan Y.H., Sun Z.W., Li J.L., Yi Y.X., Yang J., Li G.X. (2021). An electrochemical biosensor for sensitive analysis of the SARS-CoV-2 RNA. Biosens. Bioelectron..

[106] Gutierrez-Galvez L., del Cano R., Menendez-Luque I., Garcia-Nieto D., Rodriguez-Pena M., Luna M., Pineda T., Pariente F., Garcia-Mendiola T., Lorenzo E. (2022). Electrochemiluminescent nanostructured DNA biosensor for SARS-CoV-2 detection. Talanta.

[107] Forinova M., Pilipenco A., Visova I., Kuncak J., Lynn N.S., Yudin P., Dostalek J., Honig V., Palus M., Maskova H. Biosensor for rapid detection of SARS-CoV-2 in real-world samples. Proceedings of the 20th IEEE Sensors Conference.

[108] Park S., Kim H., Woo K., Kim J.M., Jo H.J., Jeong Y., Lee K.H. (2022). SARS-CoV-2 Variant Screening Using a Virus-Receptor-Based Electrical Biosensor. Nano Lett..

[109] Lee J.H., Jung Y., Lee S.K., Kim J., Lee C.S., Kim S., Lee J.S., Kim N.H., Kim H.G. (2022). Rapid Biosensor of SARS-CoV-2 Using Specific Monoclonal Antibodies Recognizing Conserved Nucleocapsid Protein Epitopes. Viruses.

[110] Kim H.Y., Lee J.H., Kim M.J., Park S.C., Choi M., Lee W., Ku K.B., Kim B.T., Park E.C., Kim H.G. (2021). Development of a SARS-CoV-2-specific biosensor for antigen detection using scFv-Fc fusion proteins. Biosens. Bioelectron..

[111] Kim S., Ryu H., Tai S., Pedowitz M., Rzasa J.R., Pennachio D.J., Hajzus J.R., Milton D.K., Myers-Ward R., Daniels K.M. (2022). Real-time ultra-sensitive detection of SARS-CoV-2 by quasi-freestanding epitaxial graphene-based biosensor. Biosens. Bioelectron..

[112] Yakoh A., Pimpitak U., Rengpipat S., Hirankarn N., Chailapakul O., Chaiyo S. (2021). Paper-based electrochemical biosensor for diagnosing COVID-19: Detection of SARS-CoV-2 antibodies and antigen. Biosens. Bioelectron..

